# Psychosomatic health status and corresponding comorbid network analysis of college students in traditional Chinese medicine schools

**DOI:** 10.3389/fpsyt.2024.1467064

**Published:** 2024-09-20

**Authors:** Shuang Yi, Xingang Hu, Chengzhe Wang, Jieqian Ge, Zixiang Ma, Yan Zhao

**Affiliations:** ^1^ School of Traditional Chinese Medicine, Beijing University of Chinese Medicine, Beijing, China; ^2^ Internal Encephalopathy of Traditional Chinese Medicine, Dongfang Hospital of Beijing University of Chinese Medicine, Beijing, China

**Keywords:** college students, psychosomatic health, anxiety, depression, somatic symptoms, comorbidity, network analysis

## Abstract

**Introduction:**

Depression, anxiety, and somatic symptoms are highly comorbid and represent the most prevalent psychosomatic health issues. Few studies have investigated the network structure of psychosomatic symptoms among traditional Chinese medicine (TCM) students. This study aims to investigate the psychosomatic health status of college students in TCM universities, while simultaneously constructing a network structure of common somatic symptoms and psychological symptoms.

**Methods:**

Online investigation was conducted among 665 students from a university of Chinese medicine. Health Status Questionnaire, Generalized Anxiety Disorder-7 (GAD-7), and Patient Health Questionnaire-2 (PHQ-2) were used to assess the mental symptoms and physical status of participants. With the R software processing, a network model of psychosomatic symptoms was constructed. Specifically, we computed the predictability (PRE), expected influence (EI), and bridging expected influence (BEI) of each symptom. Meanwhile, the stability and accuracy of the network were evaluated using the case-deletion bootstrap method.

**Results:**

Among the participants, 277 (41.65%) subjects exhibited depressive symptoms, and 244 (36.69%) subjects showed symptoms of anxiety. Common somatic symptoms included fatigue, forgetfulness, sighing, thirst, and sweating. Within the psychosomatic symptoms network, “ worrying too much about things “, “uncontrollable worries” and “weakness” exhibited the high EI and PRE, suggesting they are central symptoms. “ Little interest or pleasure in doing things,” “ feeling down, depressed, or hopeless,” “ dyssomnia,” and “sighing” with high BEI values demonstrated that they are bridging symptoms in the comorbid network.

**Conclusion:**

The psychosomatic health status of college students in traditional Chinese medicine schools is concerning, showing high tendencies for depression, anxiety, and somatic symptoms. There exists a complex relationship between somatic symptoms and psychological symptoms among students. “ Worrying too much about things “, “uncontrollable worries” and “weakness” enable to serve as comorbid intervention targets for anxiety, depression, and somatic symptoms. Addressing “ little interest or pleasure in doing things,” “ feeling down, depressed, or hopeless,” “ dyssomnia,” and “sighing” may effectively prevent the mutual transmission between psychological and physical symptoms. The network model highlighting the potential targeting symptoms to intervene in the treatment of psychosomatic health.

## 1 Introduction

Mental health is considered as the foundation of human health in the “Mental Health Action Plan (2020-2030)” of the World Health Organization (1). Nowadays, mental health issue is the leading cause of disability and a major public health concern worldwide. Depression and anxiety are important indicators of mental health, which are closely associated with somatic symptoms. Researches indicate that patients with anxiety and depression often exhibit somatic symptoms in clinical settings (2). Usually, somatic symptoms give raise to the impairment in daily life and work, as well as a primary reason for seeking medical care. Many of these symptoms are purely subjective discomfort without organic pathology, serving as outward manifestations of impaired mental health (3). Somatic symptoms, anxiety and depression constitute the three most common psychosomatic health issues. At least one-third of individuals with somatic symptom disorders concurrently experience anxiety and depression, highlighting a high comorbidity among these conditions (4, 5). The comorbid mechanisms among anxiety, depression, and somatic symptoms need to be further investigated. Menkes et al. found that some exogenous interferons can induce depression by inhibiting serotonin synthesis, thereby leading to fatigue and somatic symptoms (such as limb pain) (6). Rudolf et al. discovered that patients with anxiety disorders exhabited lower autonomic nervous system adaptability compared to healthy individuals, with more abnormal neuroregulation. As a result, the lower perception threshold for external stimuli is obtained for the patients, causing their central nervous system to struggle in accurately distinguishing whether the received stimuli are related to anxiety or neutral stimuli (7). Meanwhile, research shows a gradual increase in psychosomatic health issues among adolescents (8, 9).The somatic and psychological problems has became the significant components of mental disorders (10).

Current research on anxiety, depression, and somatic symptoms mostly relies on traditional latent variable theories in which the symptoms of mental disorders are interpreted as outcomes of underlying common factors (11). However, from this perspective, the co-occurrence or random clustering of different symptoms in mental disorders is attributed to the latent common factors that cannot be directly observed (12). Therefore, these methods based on the latent variable theories usually capture common differences among all symptoms. They overlook information related to the individual development of mental disorders (13, 14).

Recently, the network analysis has provided a new insight to understanding psychopathological symptoms (15). The network theory of psychopathology no longer regards mental disorders as underlying entities behind symptoms, but rather considers symptoms as integral components of mental disorders (16). It explores the interactions among individual psychopathological symptoms to reveal connections among individual variables (15, 17). In network analysis, nodes represent symptoms, and edges (lines between nodes) denote connections between symptoms. The weight of an edge signifies the strength of the association. The nodes connected with more edges and with larger weights suggest their higher centrality (18). Researchers often focus on nodes with higher centrality because these nodes can bring about prominent influence or can be used to predict other nodes. Additionally, network analysis offers a fresh perspective on the mechanisms of comorbidity in mental disorders, providing an intuitive depiction of the relationships among symptom clusters (19, 20). The “bridge variables” are established to connect different symptom clusters, which are beneficial to understand interactions between symptom clusters and identify targets for targeted interventions (20–22). Numbers of studies have utilized network analysis in mental disorders. Yang et al., explored the correlation among personality traits, anxiety and depression in college students (23). Luo et al., analyzed the comorbid characteristics of anxiety and depression symptoms in the student groups (14). Liu et al., used network analysis to explore bridging symptoms between depression and anxiety in HIV patients (24). However, the network analysis between psychosomatic symptoms needs to be further explored. Constructing networks of psychosomatic symptoms allows exploration of the relationship between somatic symptoms and psychological symptoms from a comorbidity perspective, thereby bridging research gaps between physical and psychological fields.

College students are a critical transitional stage from late adolescence to early adulthood, which are a high-risk group for physical and mental illnesses. Compared to other countries, the incidence of psychosomatic health issues among Chinese university students is relatively high (25–27), which may be owing to the large population, significant competitive pressure, and limited resources for mental health education. Among undergraduate students in Chinese comprehensive universities, 11.8% of them exhibited severe or moderate somatic symptoms; the students with severe anxiety symptoms accounted for 7.8% of the surveyed students; and severe depression symptoms are reported by 23.3% (28). Medical schools are a relatively unique category within universities, characterized by specialized programs, longer durations, extensive coursework, and high employment pressures. These characteristics of medical schools contribute to greater stress for medical students. Research suggested that 20% to 67% of medical students experience varying degrees of psychosomatic health issues, which was significantly higher than the 10% to 30% observed in regular college students (29).Within medical schools, Traditional Chinese Medicine (TCM) universities represent a unique presence because of blending elements of medical education with both natural and humanistic sciences. Meanwhile, TCM universities are widespread across nearly every province of China. However, there is currently limited research on the psychosomatic health characteristics of college students in TCM universities.

In this work, taking the TCM college students as objects, their psychosomatic status was investigated by a comprehensive questionnaire survey. Based on the investigation results, we constructed a network model (Psychosomatic Symptoms Network Model) which comprised somatic symptoms and anxiety-depression symptoms. The central and bridging symptoms within this network were also explored to elucidate important connections between somatic symptoms and psychological symptoms. This work describes the current psychosomatic health status of TCM college students. In addition, the network model we proposed provides theoretical insights into specific pathways linking somatic symptoms with psychological symptoms.

## 2 Materials and methods

### 2.1 Participants

An online survey was adopted via the survey links (www.wjx.cn), and the survey link was distributed to college students from Beijing University of Chinese Medicin. Participants were briefed on the purpose of survey and how to complete the questionnaire. The informed consents were obtained from participants. A total of 665 participants completed the survey from April to May 2024. The survey consisted of conventional information of the participant, Health Status Questionnaire, Generalized Anxiety Disorder-7 (GAD-7), and Patient Health Questionnaire-2 (PHQ-2). This work strictly adhered to the principles of “Helsinki Declaration” and received approval from the Ethics Committee of BUCM (No. 2024BZYLL0105)

### 2.2 Measures

#### 2.2.1 Health status questionnaire

The health status questionnaire was revised based on literature research and expert consultation. It is a self-assessment questionnaire that includes 30 relevant symptoms (items) categorized into overall symptoms, head-face-neck symptoms, chest-abdomen symptoms, diet, sleep, bowel movements, etc. All items are presented in clear and understandable description. Each item employs a 4-point rating scale: 0 points for “none,” 1 point for “occasional,” 2 points for “sometimes,” and 3 points for “frequent”. For example, “Do you feel dizzy?” Responses such as “none,” “occasional,” “sometimes,” and “frequent” correspond to 0 points, 1 point, 2 points, and 3 points respectively. Previous research has confirmed that this questionnaire has good reliability and validity, accurately reflecting the participants’ physical health status (30). In this investigation, the Cronbach’s α for health status questionnaire was 0.92.

#### 2.2.2 Generalized anxiety disorder-7

The GAD-7 is a common tool for evaluating anxiety symptoms, developed by Spitzer et al (31). This investigation employed the version of GAD-7 revised by He et al. The version is suitable for the Chinese context and has demonstrated good reliability and validity among Chinese populations (32, 33). The GAD-7 consists of 7 items including excessive worry, difficulty relaxing, feeling restless, irritability, and fear. Each item employs a 4-point Likert scale: 0, 1, 2, 3 points representing “not at all,” “several days,” “more than half the days,” and “nearly every day,” respectively. A total score of ≥ 5 on the GAD-7 indicates an anxiety state (34). In this investigation, the Cronbach’s α for the GAD-7 was 0.91.

#### 2.2.3 Patient health questionnaire-2

The PHQ-2, developed by Kroenke et al., is a brief and widely used screening tool for depression (35). The PHQ-2 consists of 2 items: “little interest or pleasure in doing things” and “feeling down, depressed, or hopeless”. Similarly, a 4-point Likert scale was employed for each item: 0, 1, 2, 3 points indicating “not at all,” “several days,” “more than half the days,” and “nearly every day,” respectively. A total score of ≥ 2 on the PHQ-2 suggests the depression (36). Related studies have confirmed that the PHQ-2 has good reliability and validity in screening for depressive symptoms (36). In this investigation, the Cronbach’s α for the PHQ-2 was 0.72.

### 2.3 Statistical analysis

#### 2.3.1 General information statistics

Descriptive statistics of the participants were conducted by SPSS 26.0. The classification data was expressed in terms of frequencies and component ratios. The mean ± standard deviation was used for the description of continuous variables. Bivariate correlations between psychological symptoms and somatic symptoms were obtained by Spearman correlation analysis.

#### 2.3.2 Network model construction

The qgraph package in R software (version 4.4.0) (37) was employed to construct symptom networks based on EBICglasso function and Spearman correlation analysis. The EBICglasso function combines the least absolute shrinkage and selection operator (LASSO) regularization with extended Bayesian information criterion (EBIC) (23). In this work, the EBIC hyperparameter γ was set to 0.5 (19). The Fruchterman-Reingold layout was utilized (38). The network was divided into the somatic symptom community and the anxiety-depression community. Nodes in each community represent somatic symptoms or items from GAD-7 and PHQ-2 scales. In the visualized network, blue edges between nodes indicate positive correlations, while red edges indicate negative correlations. Thicker edges signify stronger correlations between adjacent nodes (39).

The expected influence (EI) of each node was also calculated by qgraph package, which sums the values of all edges connected to that specific node. A higher EI value demonstrates the greater importance of the node within the network (40). Meanwhile, the bridge expected influence (BEI) of each node is calculated to identify bridging symptoms (41). The BEI is the sum of edge weights between a specific node and nodes in other communities (20). A larger BEI suggests the stronger influence of that node on another community (41). Nodes can be forecasted through their neighboring nodes. Predictability (PRE) of each node was obtained by R-package mgm (42). With PRE ranging from 0 to 1, the node with higher PRE indicates the stronger predictive ability of this node (43).

Network robustness test were assessed using the bootnet package, which includes the stability and the accuracy of the network (19). With a non-parametric bootstrap (1000 bootstrap samples), the accuracy of edge weight was evaluated via computing 95% confidence intervals (CI). Case-deletion bootstrap was employed to calculate the stability coefficient, with coefficients above 0.5 to indicate good stability of centrality indices (19). Bootstrap difference tests (1000 bootstrap samples, α = 0.05) were employed on edge weights, EI, and BEI to examine differences between two edge weights or two nodes.

## 3 Results

### 3.1 Demographic characteristics and descriptive statistics

Among 665 participants, there were 193 males (29.02%) and 472 females (70.98%). The age ranged from 18 to 32 years old, with an average age of 22.38 ± 3.20 years. Among them, 443 (66.62%) participants were undergraduates and 222 (33.38%) participants were graduate students.

#### 3.1.1 Mental health

Varying degrees of anxiety and depression were discovered among participants, with 244 (36.69%) experiencing anxiety and 277 (41.65%) experiencing depression. Based on the GAD-7 and PHQ-2, the total scores, mean (M), standard deviation (SD), as shown in **Table 1**. Node numbers, Pre, EI, and BEI of items were also listed **Table 1**.

**Table 1:** Item’s total score, M, SD, Pre, EI, BEI values.

Item	Number	M ± SD	PRE	EI	BEI
**PHQ-2 total score**		1.25 ± 1.19			
Little interest or pleasure in doing things	P1	0.59 ± 0.70	0.54	0.57	0.42
Feeling down, depressed, or hopeless	P2	0.66 ± 0.65	0.63	0.83	0.42
**GAD-7 total score**		3.86 ± 4.31			
Feeling nervous, anxious or eager	G1	0.80 ± 0.69	0.59	0.05	0.28
Uncontrollable worries	G2	0.56 ± 0.81	0.67	1.61	0.22
Worrying too much about things	G3	0.65 ± 0.83	0.69	1.51	0.11
Trouble relaxing	G4	0.60 ± 0.83	0.6	0.71	0.17
Unable to sit still due to restlessness	G5	0.37 ± 0.69	0.52	-0.71	0.17
Easily annoyed or irritable	G6	0.53 ± 0.77	0.59	0.39	0.21
Feeling afraid as if something awful might happen	G7	0.37 ± 0.68	0.54	-0.82	0.13

#### 3.1.2 Somatic symptoms

About 30 somatic symptoms were investigated, and their frequencies were studied, as shown in **Table 2**. Common somatic symptoms (> 50%) among participants included fatigue, forgetfulness, sighing, thirst, and sweating in order of frequency.

**Table 2:** Somatic symptoms and corresponding frequency.

Item	Frequency	Item	Frequency	Item	Frequency
fatigue	82.86%	pant	44.36%	soreness of waist	35.49%
forgetfulness	73.38%	eating disorder	43.91%	fever	35.34%
sighing	68.27%	bellyache	41.81%	cough	31.88%
thirst	61.96%	xerophthalmia	40.45%	foreign body sensation	30.08%
sweating	56.24%	headache	40.30%	anal burning	28.87%
stool abnormity	47.22%	stomach distension	38.64%	abdominal distension	28.57%
dyssomnia	47.21%	chest distress	37.90%	frequent micturition	28.12%
weakness	46.47%	dizzy	37.59%	nausea	25.71%
lumbago	46.32%	cold	36.69%	tinnitus	24.66%
alopecia	46.17%	palpitation	35.94%	hiccup	23.60%

The correlation between somatic symptoms and anxiety/depression was shown in **Table 3**. Symptoms such as fatigue, forgetfulness, sighing, stool abnormity, dyssomnia, weakness, pant, stomach distension, chest tightness, palpitations, and soreness of waist had correlation coefficients r ≥ 0.5 with depression or anxiety, indicating strong relationships among these variables. Moreover, all symptoms were analyzed in the comorbid network to calculate their scores (M, SD) and parameters (PRE, EI, and BEI), as outlined in **Table 4**.

**Table 3:** Correlation among somatic symptoms, depression, and anxiety.

Item	Depression	Anxiety
total score	0.798*	0.717*
fatigue	0.573*	0.531*
forgetfulness	0.504*	0.468*
sigh	0.598*	0.575*
stool abnormity	0.510*	0.423*
dyssomnia	0.691*	0.551*
weakness	0.599*	0.536*
pant	0.519*	0.459*
stomach distension	0.500*	0.474*
chest distress	0.588*	0.539*
palpitation	0.509*	0.487*
soreness of waist	0.539*	0.491*
thirst	0.481*	0.478*
sweat	0.492*	0.489*
lumbago	0.452*	0.417*
alopecia	0.368*	0.351*
eating disorder	0.455*	0.381*
bellyache	0.388*	0.370*
xerophthalmia	0.388*	0.344*
headache	0.489*	0.418*
dizzy	0.461*	0.426*
cold	0.310*	0.314*
fever	0.332*	0.296*
cough	0.290*	0.263*
foreign body sensation	0.308*	0.278*
anal burning	0.375*	0.303*
abdominal distension	0.456*	0.425*
frequent micturition	0.399*	0.389*
nausea	0.405*	0.382*
tinnitus	0.323*	0.261*
hiccup	0.409*	0.390*

* means P < 0.05, which has statistical significance.

**Table 4:** Item’s M, SD, Pre, EI, BEI values.

Item	Number	M ± SD	Pre	EI	BEI
fatigue	S1	1.45 ± 1.06	0.56	0.77	0.14
forgetfulness	S2	1.20 ± 1.03	0.44	-1.15	0.09
sighing	S3	1.00 ± 0.93	0.59	0.46	0.39
stool abnormity	S4	0.67 ± 0.84	0.28	-1.88	0.17
dyssomnia	S5	0.69 ± 0.88	0.42	-1.34	0.46
weakness	S6	0.66 ± 0.94	0.57	1.15	0.18
pant	S7	0.62 ± 0.83	0.41	-0.72	0.1
stomach distension	S8	0.54 ± 0.79	0.35	-1.18	0.13
chest distress	S9	0.51 ± 0.79	0.53	0.47	0.21
palpitation	S10	0.45 ± 0.69	0.43	-0.53	0.15
soreness of waist	S11	0.49 ± 0.77	0.49	-0.19	0.13

### 3.2 Network structure

**Figure 1:**
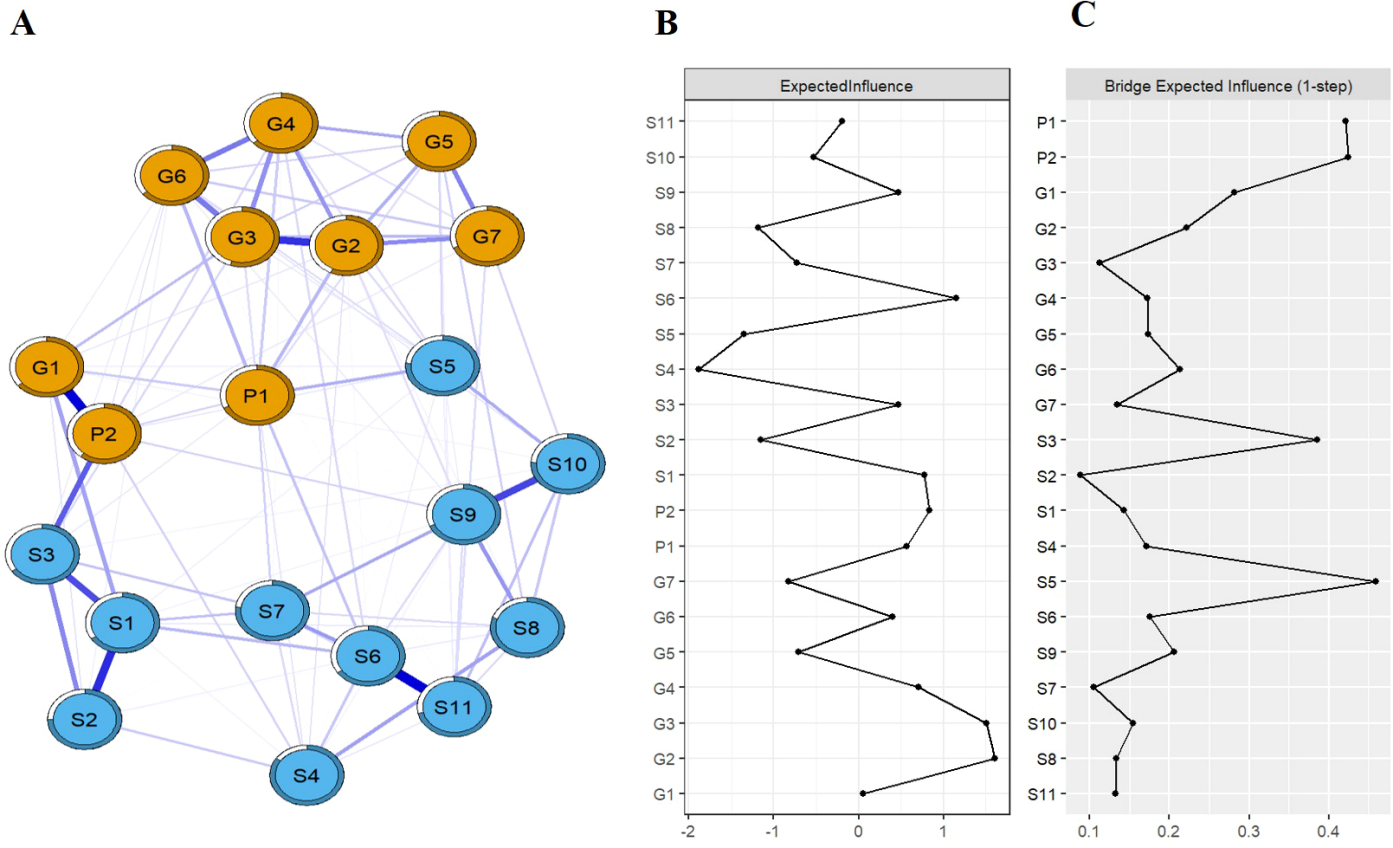
Psychosomatic symptom network structure and node EI/BEI value.

**Figure 1A** depicts the network structure of somatic symptoms with anxiety-depressive symptoms. The network structure (average weight of 0.048) includes 129 non-zero edges, and edge weights range from 0.00 to 0.38. Within the network, 52 edges (40.31%) bridge the somatic symptoms and the symptoms of anxiety/depression, and these52 bridging edges are all positively correlated. These edges with the top three weights are the bridge between “Feeling down, depressed, or hopeless” and “sighing” (edge weight = 0.24), the bridge between “Feeling nervous, anxious or eager” and “fatigue” (edge weight = 0.14), and the bridge between “Little interest or pleasure in doing things” and “dyssomnia” (edge weight = 0.12). The correlation matrix of the network is also shown in **Supplementary Table S1** of **Supplementary Material**. Bootstrap estimation of edge weights shows relatively narrow CI, indicating reliable evaluation of these edge weights, as depicted in **Supplementary Figure S1**. Testing differences of edge are also shown in **Supplementary Figure S2**. Additionally, predictability of node is represented by circle around this node. PRE values of nodes range from 0.28 to 0.69 in this network. The nodes of “Uncontrollable worries” and “Worrying too much about things” exhibit the high predictability, indicating that 67% and 69% of their variances can be explained by adjacent nodes, respectively.

The expected influences among somatic manifestations and symptoms of anxiety/depression are shown in **Figure 1B**. In the network, the nodes of “Uncontrollable worries”, “Worrying too much about things”, and “weakness” exhibit the large EI values (1.61, 1.51, and 1.15, respectively). Statistically, these three symptoms have the highest associations in comorbid network, considered as the central symptoms. Bootstrap difference test reveals a stable coefficient of 0.75 for the EI, suggesting the stability of EI evaluation (**Supplementary Figure S3**). Furthermore, bootstrap difference tests of EI demonstrate significant differences among the central symptoms and the majority (≥50%) of other symptoms, as shown in **Supplementary Figure S4**.

The bridging expected influences among somatic manifestations and symptoms of anxiety/depression are depicted in **Figure 1C**. Larger BEI values indicate stronger bridging centrality. The nodes of “dyssomnia”, “Little interest or pleasure in doing things”, “Feeling down, depressed, or hopeless”, and “sighing” are identified as bridging symptoms because of their high BEI (0.46, 0.42, 0.42, and 0.39, respectively). Bootstrap difference test obtains a coefficient of 0.67 for the stability of BEI evaluation (**Supplementary Figure S5**). Meanwhile, bootstrap difference tests of BEI demonstrate significant differences among the bridging symptoms and most other nodes (**Supplementary Figure S6**).

## 4 Discussion

In this study, the proportion of depression among college students was 41.65%, and anxiety was 36.69%. These proportions are significantly higher than the average rates of 32.74% for depression and 27.22% for anxiety among medical students in China, respectively (44). The differences of proportions may be attributed to variations of subjects, measurement tools, methods, and geographical factors. The high proportions reflect the serious states of depression and anxiety among college students of TCM universities. Compared to general medical students, TCM students face unique challenges such as learning both TCM and Western medicine, high academic pressures, and intense competition in employment. With the challenges exceeding their coping abilities, they are more prone to developing mental health issues (i.e. depression and anxiety). For physical health, we found various somatic symptoms with high frequencies (> 20%) among college students. Furthermore, the frequencies of some somatic manifestations (such as fatigue, forgetfulness, sighing, thirst, and sweating) exceed 50%. These physical discomforts have become prevalent issues affecting academic performance and daily lives of students, demanding significant attention.

From a causal systems perspective (CSP), this study investigated the interactions among psychosomatic symptoms in TCM university students. Compared with the traditional common cause perspective (CCP), this work attributed comorbidities to direct interactions among symptoms, providing an alternative explanation (14). The work identified the symptoms of “ worrying too much about things “, “uncontrollable worries” and “weakness” as central symptoms in the network. Both symptoms of “uncontrollable worries” and “ worrying too much about things “ refer to a persistent state of anxiousness, which are the common central symptoms in existing anxiety and depression network models. Cai et al. reported that the symptom of “worrying too much about things” was the central symptom of the anxiety-depression network adolescents in the later stages of the COVID-19 pandemic (45). Zhang et al. discovered that the symptoms of “uncontrollable worries” and “worrying too much about things” were the core symptoms in the anxiety-depression network of Chinese elderly diabetic patients (46). Similar results have been reported in investigations on new university students of China (14). The recurring presence of “uncontrollable worries” or “worrying too much about things” as the central symptom across different groups highlights the significance in psychological manifestations. With insighting into the neuroendocrine perspective, persistent worry results in overactivity of hypothalamic-pituitary-adrenal (HPA) axis, increasing cortisol secretion, thereby leading to a range of somatic symptoms (i.e. dizziness, headaches, fatigue and palpitations) (47, 48). Therefore, the modulation of unnecessary worry is crucial for improving the psychosomatic health of college students. As another central symptom in this network, the somatic manifestation of “weakness” means a persistent feeling of tiredness and lack of energy. This sensation can significantly impact the activities in daily life, which also exhibits a relatively high occurrence (46.47%) among college students. College students often face high academic demands, such as complex subject knowledge, heavy assignments, and exam tasks. This pressure can lead to long study hours, insufficient rest and relaxation, ultimately resulting in weakness. Weakness can be classified into physiological and pathological types. Physiological weakness is usually caused by factors such as excessive exertion, lack of sleep, or poor nutrition, while pathological weakness may be a symptom of certain diseases. Psychological disorders (especially depression and anxiety) often accompany weakness, which affects not only the body but also emotional states. Additionally, weakness also contributes to psychological health issues. Chronic tiredness may lead to mood disturbances, anxiety, and depression, thereby creating a vicious cycle. From a psychopathological perspective, the comorbid mechanisms of weakness and psychological disorders involve complex interactions among neurobiological, inflammatory, sleep quality, and psychosocial factors. Within the comorbid network, these three symptoms are strongly associated with other symptoms because of their high EI values, playing a crucial role in activating and maintaining the network. Intervening in central symptoms of network can effectively reduce the overall severity of symptoms, thereby promoting treatment and prevention (49, 50).

BEI is an indicator for identifying bridging symptoms, and in this study the four symptoms with the highest BEI values were identified: “little interest or pleasure in doing things”, “feeling down, depressed, or hopeless”, “sighing” and “dyssomnia”. In network analysis, bridging symptoms have cross-diagnostic significance as they serve to connect symptom networks from two different communities. Although there is a lack of network analysis studies on psychosomatic symptoms, in anxiety-depression network models, symptom of “feeling down, depressed, or hopeless”and “little interest or pleasure in doing things” appears as bridging symptoms among Chinese new college students (14). During the COVID-19 pandemic, symptoms of “little interest or pleasure in doing things” and “feeling down, depressed, or hopeless” were identified as bridging symptoms in the anxiety-depression network of nursing students (51). Additionally, “little interest or pleasure in doing things” and “feeling down, depressed, or hopeless” were also typical symptoms for diagnosing major depression (52). The above studies highlight the importance of bridging symptoms “little interest or pleasure in doing things” and “feeling down, depressed, or hopeless” in psychological clinical manifestations, which matches the findings in this network analysis. In this study, these two symptoms also demonstrated their significant impact on physical manifestations, specifically their strong capacity to increase the risk of somatic symptoms. The somatic symptoms of “sighing” and “dyssomnia” are two additional bridging symptoms identified in this network, which exhibit the strongest ability to increase the risk of anxiety and depression contagion. Sighing is an external manifestation of anxiety/depression, and individuals may sigh to alleviate inner tension and repression when feeling anxious or depressed. However, frequent sighing enables to lead individuals to focus more on their negative emotions, worsening anxiety and depression. The serious influence makes sighing an important link in psychosomatic symptoms. Another bridging symptom is “ dyssomnia,” which aligns with previous research findings. A systematic review indicates that dyssomnia is bidirectionally associated with anxiety and depression in adolescents, adults, and the elderly (53). Meanwhile, the somatic manifestations of “dyssomnia” were also identified as a bridging symptom in the network of anxiety, depression, and insomnia for clinical practitioners with depressive symptoms (54). In terms of psychopathology, brain neurotransmitters are considered a common underlying mechanism linking dyssomnia with depression/anxiety. Imbalances in neurotransmitters such as norepinephrine, serotonin, and dopamine can affect mood and sleep. Furthermore, a lack of sleep can reversely disrupt these neurotransmitters, yielding a negative feedback loop. As mentioned, bridging symptoms play a crucial role in the generation of comorbidities, which give raise to the onset and persistence of mental comorbidities. Intervening in bridging symptoms can effectively prevent or alleviate comorbid symptoms (55, 56).

The average node predictability of the network is 0.52, indicating a moderate level of self-determination (43). The predictability of nodes can reflect the controllability of the network and determine the effectiveness of the planned treatment (43). In this study, “uncontrollable worry” and “ Worrying too much about things” showed high predictability values that could be controlled by intervening in their adjacent nodes. However, symptoms with lower predictability, like “ stool abnormity” and “ stomach distension” may require direct control or intervention from external factors outside the network (39).

The study exhibits limitations. The investigation employed convenience sampling from a single TCM university with an uneven gender ratio. Recruiting participants from different regions on a larger scale will be considered. Meanwhile, it is also a cross-sectional study, preventing examination of dynamic or causal relationships among symptoms. Future longitudinal studies should explore these relationships. In addition, the use of brief scales (PHQ-2 and GAD-7) may have limited the ability to capture the full spectrum of psychological symptoms. The study found that the effect between somatic symptoms and psychological symptoms was relatively weak, which may indicate that the actual effect is limited. Future intervention trials targeting central and bridging symptoms are needed to validate its effectiveness.

This work investigated the psychosomatic health status of college students of TCM, thereby establishing a network model of psychosomatic symptoms. Within the comorbid network, the centrality, bridging role, and predictability of symptoms were explored. We found that the psychosomatic health of these students is concerning, showing tendencies towards high levels of depression, anxiety, and somatization symptoms. After comorbid network analysis, the symptoms of “ worrying too much about things “, “uncontrollable worries” and “weakness” were identified as central symptoms in the network model. Targeting these central symptoms for intervention could further relieve overall somatic presentations and reduce the severity of anxiety or depression. Simultaneously, interventions of targeting nodes with high predictability (“uncontrollable worry” and “ Worrying too much about things”) can be achieved by intervening in their adjacent nodes. Bridging symptoms (“ little interest or pleasure in doing things,” “ feeling down, depressed, or hopeless,” “ dyssomnia,” and “sighing”) can effectively prevent or alleviate the symptoms of comorbidity. This study will serve as a reference for psychosomatic health interventions among college students in TCM universities.

## Data Availability

The raw data supporting the conclusions of this article will be made available by the authors, without undue reservation.
