# Supplementary figures and images for: Psychosomatic health status and corresponding comorbid network analysis of college students in traditional Chinese medicine schools
Source: Front Psychiatry. 2024 Sep 20;15:1467064. doi: 10.3389/fpsyt.2024.1467064 (PMC11450477; doi:10.3389/fpsyt.2024.1467064)

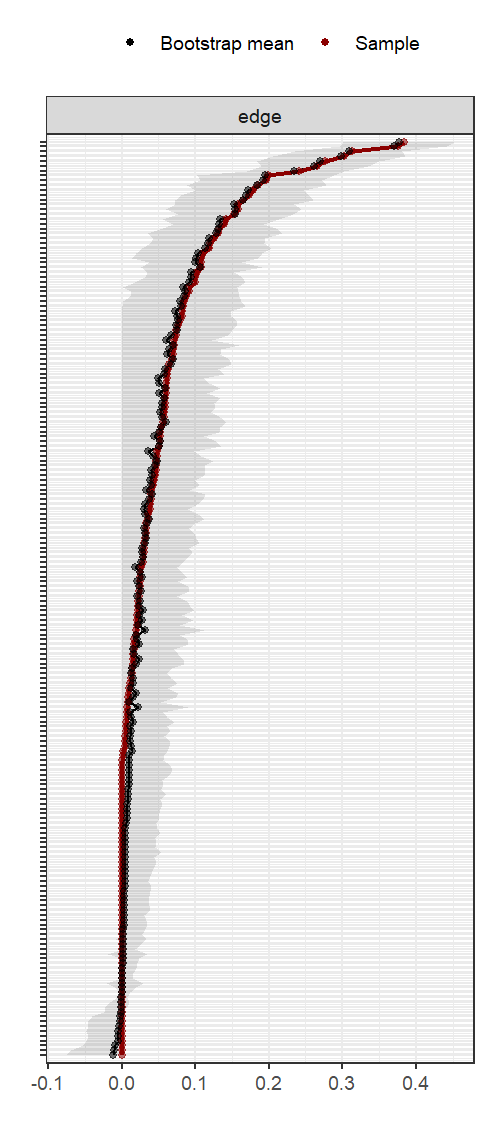

Supplement: Supplementary file 1 [file Image1.png]

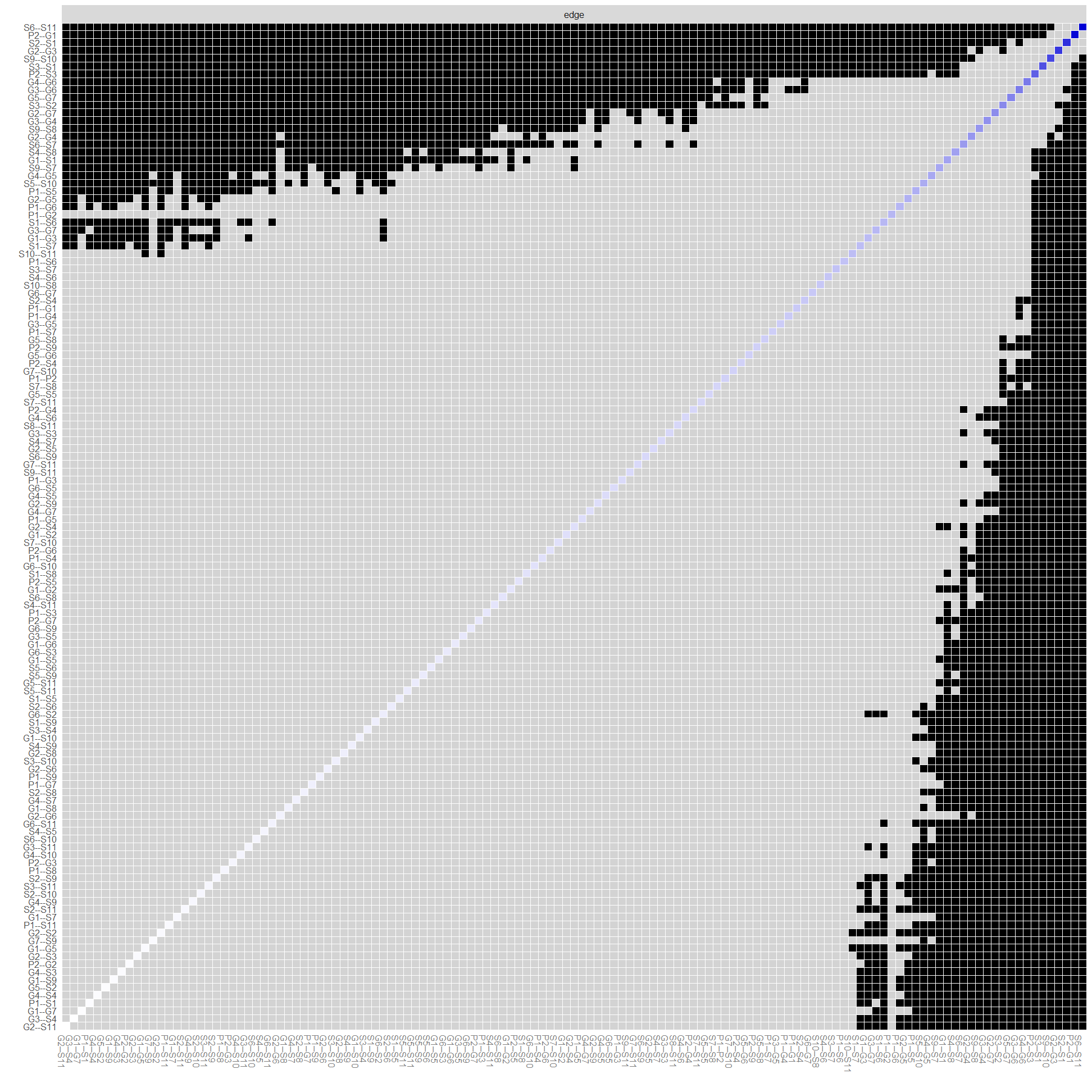

Supplement: Supplementary file 2 [file Image2.png]

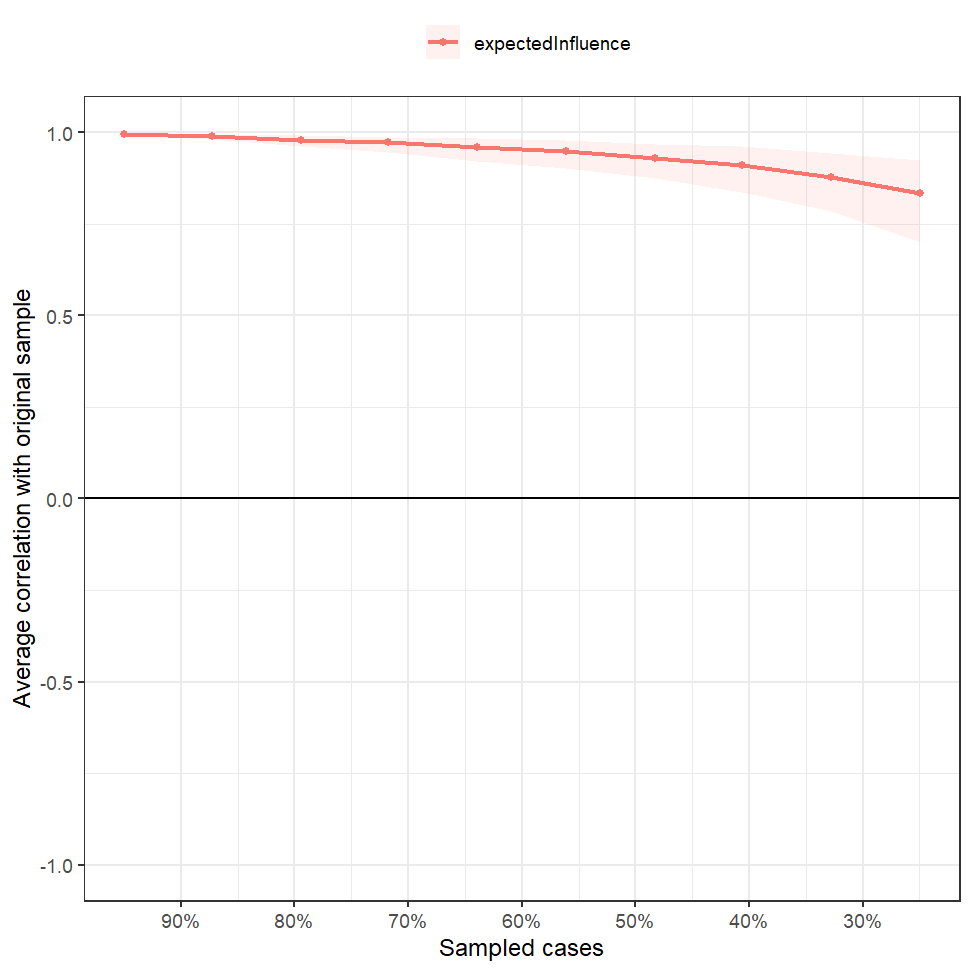

Supplement: Supplementary file 3 [file Image3.png]

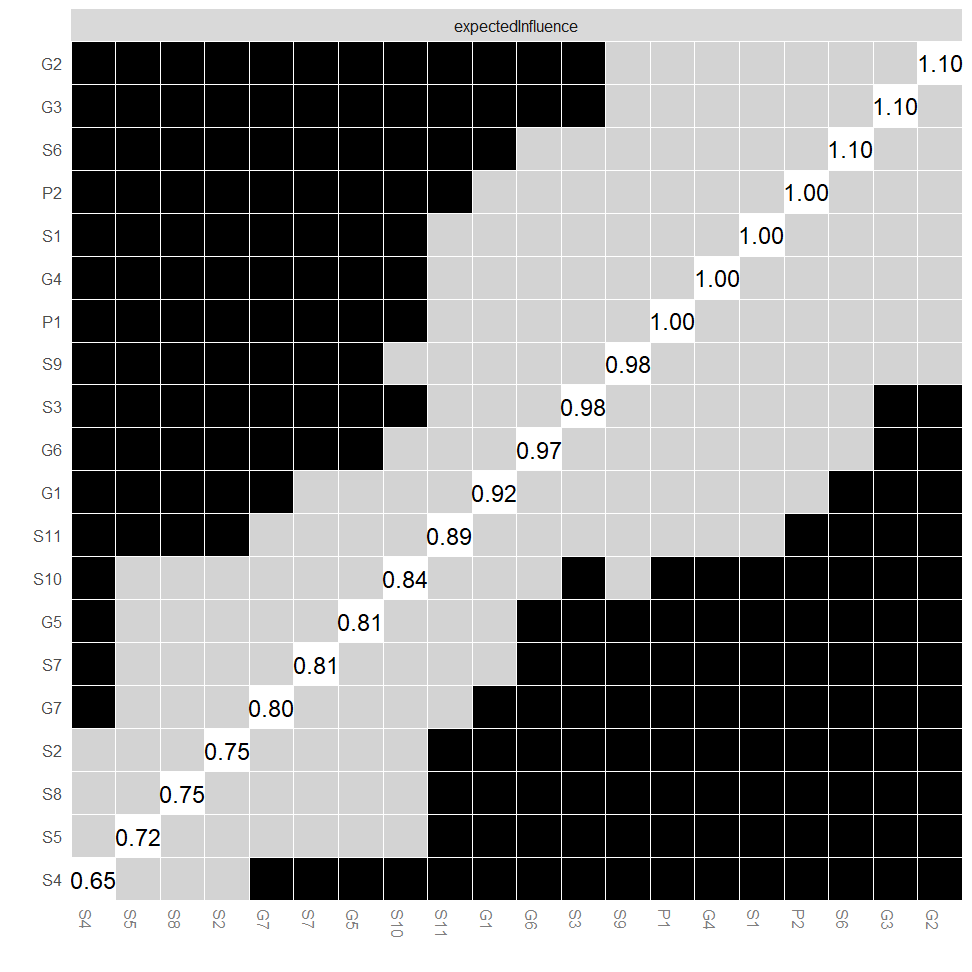

Supplement: Supplementary file 4 [file Image4.png]

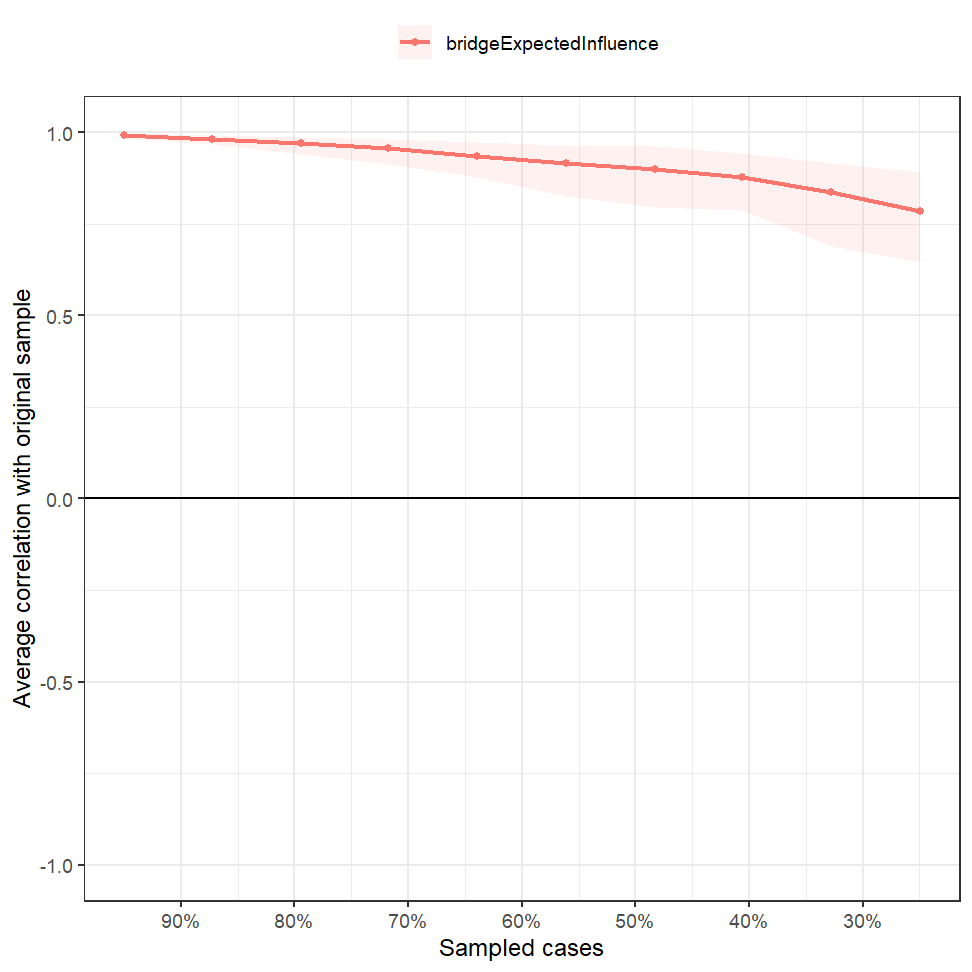

Supplement: Supplementary file 5 [file Image5.png]

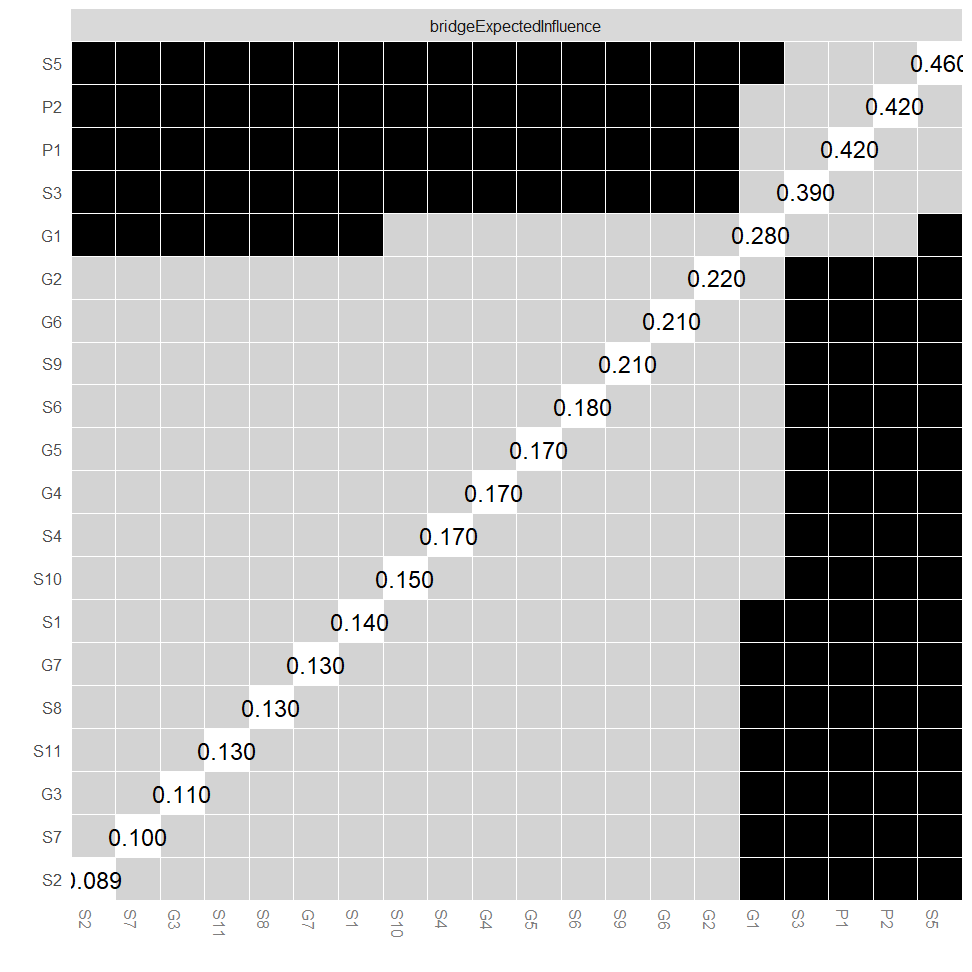

Supplement: Supplementary file 6 [file Image6.png]
