# Supplementary material for: Psychosomatic health status and corresponding comorbid network analysis of college students in traditional Chinese medicine schools
Source: Front Psychiatry. 2024 Sep 20;15:1467064. doi: 10.3389/fpsyt.2024.1467064 (PMC11450477; doi:10.3389/fpsyt.2024.1467064)
Supplement: Supplementary file 7 [file Table1.docx]

Table S1 The Correlation Matrix of the Psychosomatic health Network

|  | P1 | P2 | G1 | G2 | G3 | G4 | G5 | G6 | G7 | S3 | S2 | S1 | S4 | S5 | S6 | S9 | S7 | S10 | S8 | S11 |
| --- | --- | --- | --- | --- | --- | --- | --- | --- | --- | --- | --- | --- | --- | --- | --- | --- | --- | --- | --- | --- |
| P1 |  | 0.069 | 0.082 | 0.108 | 0.056 | 0.080 | 0.051 | 0.111 | 0.022 | 0.038 | 0.000 | 0.004 | 0.047 | 0.120 | 0.092 | 0.022 | 0.074 | 0.000 | 0.015 | 0.008 |
| P2 | 0.069 |  | 0.376 | 0.006 | 0.015 | 0.061 | 0.000 | 0.047 | 0.038 | 0.242 | 0.000 | 0.000 | 0.069 | 0.042 | 0.000 | 0.072 | 0.000 | 0.000 | 0.000 | 0.000 |
| G1 | 0.082 | 0.376 |  | 0.040 | 0.102 | 0.000 | 0.007 | 0.034 | 0.002 | 0.000 | 0.048 | 0.139 | 0.000 | 0.033 | 0.000 | 0.005 | 0.010 | 0.024 | 0.021 | 0.000 |
| G2 | 0.108 | 0.006 | 0.040 |  | 0.302 | 0.158 | 0.119 | 0.019 | 0.173 | 0.007 | 0.008 | 0.000 | 0.049 | 0.059 | 0.023 | 0.052 | 0.000 | 0.000 | 0.024 | 0.000 |
| G3 | 0.056 | 0.015 | 0.102 | 0.302 |  | 0.166 | 0.076 | 0.197 | 0.107 | 0.060 | 0.000 | 0.000 | 0.001 | 0.036 | 0.000 | 0.000 | 0.000 | 0.000 | 0.000 | 0.016 |
| G4 | 0.080 | 0.061 | 0.000 | 0.158 | 0.166 |  | 0.131 | 0.200 | 0.052 | 0.006 | 0.000 | 0.000 | 0.005 | 0.052 | 0.061 | 0.012 | 0.021 | 0.016 | 0.000 | 0.000 |
| G5 | 0.051 | 0.000 | 0.007 | 0.119 | 0.076 | 0.131 |  | 0.071 | 0.186 | 0.000 | 0.005 | 0.000 | 0.000 | 0.063 | 0.000 | 0.000 | 0.000 | 0.000 | 0.074 | 0.031 |
| G6 | 0.111 | 0.047 | 0.034 | 0.019 | 0.197 | 0.200 | 0.071 |  | 0.083 | 0.034 | 0.027 | 0.000 | 0.000 | 0.053 | 0.000 | 0.036 | 0.000 | 0.045 | 0.000 | 0.019 |
| G7 | 0.022 | 0.038 | 0.002 | 0.173 | 0.107 | 0.052 | 0.186 | 0.083 |  | 0.000 | 0.000 | 0.000 | 0.000 | 0.000 | 0.000 | 0.008 | 0.000 | 0.069 | 0.000 | 0.058 |
| S3 | 0.038 | 0.242 | 0.000 | 0.007 | 0.060 | 0.006 | 0.000 | 0.034 | 0.000 |  | 0.178 | 0.265 | 0.025 | 0.000 | 0.000 | 0.000 | 0.092 | 0.024 | 0.000 | 0.014 |
| S2 | 0.000 | 0.000 | 0.048 | 0.008 | 0.000 | 0.000 | 0.005 | 0.027 | 0.000 | 0.178 |  | 0.314 | 0.083 | 0.000 | 0.029 | 0.014 | 0.000 | 0.013 | 0.021 | 0.010 |
| S1 | 0.004 | 0.000 | 0.139 | 0.000 | 0.000 | 0.000 | 0.000 | 0.000 | 0.000 | 0.265 | 0.314 |  | 0.000 | 0.029 | 0.108 | 0.026 | 0.100 | 0.000 | 0.044 | 0.000 |
| S4 | 0.047 | 0.069 | 0.000 | 0.049 | 0.001 | 0.005 | 0.000 | 0.000 | 0.000 | 0.025 | 0.083 | 0.000 |  | 0.017 | 0.087 | 0.024 | 0.059 | 0.000 | 0.141 | 0.040 |
| S5 | 0.120 | 0.042 | 0.033 | 0.059 | 0.036 | 0.052 | 0.063 | 0.053 | 0.000 | 0.000 | 0.000 | 0.029 | 0.017 |  | 0.031 | 0.031 | 0.000 | 0.126 | 0.000 | 0.030 |
| S6 | 0.092 | 0.000 | 0.000 | 0.023 | 0.000 | 0.061 | 0.000 | 0.000 | 0.000 | 0.000 | 0.029 | 0.108 | 0.087 | 0.031 |  | 0.058 | 0.153 | 0.017 | 0.040 | 0.384 |
| S9 | 0.022 | 0.072 | 0.005 | 0.052 | 0.000 | 0.012 | 0.000 | 0.036 | 0.008 | 0.000 | 0.014 | 0.026 | 0.024 | 0.031 | 0.058 |  | 0.133 | 0.277 | 0.158 | 0.058 |
| S7 | 0.074 | 0.000 | 0.010 | 0.000 | 0.000 | 0.021 | 0.000 | 0.000 | 0.000 | 0.092 | 0.000 | 0.100 | 0.059 | 0.000 | 0.153 | 0.133 |  | 0.047 | 0.064 | 0.061 |
| S10 | 0.000 | 0.000 | 0.024 | 0.000 | 0.000 | 0.016 | 0.000 | 0.045 | 0.069 | 0.024 | 0.013 | 0.000 | 0.000 | 0.126 | 0.017 | 0.277 | 0.047 |  | 0.085 | 0.099 |
| S8 | 0.015 | 0.000 | 0.021 | 0.024 | 0.000 | 0.000 | 0.074 | 0.000 | 0.000 | 0.000 | 0.021 | 0.044 | 0.141 | 0.000 | 0.040 | 0.158 | 0.064 | 0.085 |  | 0.060 |
| S11 | 0.008 | 0.000 | 0.000 | 0.000 | 0.016 | 0.000 | 0.031 | 0.019 | 0.058 | 0.014 | 0.010 | 0.000 | 0.040 | 0.030 | 0.384 | 0.058 | 0.061 | 0.099 | 0.060 |  |

Note: P1=Little interest or pleasure in doing things; P2=Feeling down, depressed, or hopeless; G1=Feeling nervous, anxious or eager; G2=Uncontrollable worries; G3=Worrying too much about things;G4=Trouble relaxing;G5=Unable to sit still due to restlessness;G6=Easily annoyed or irritable;G7=Feeling afraid as if something awful might happen; S1=fatigue;S2=forgetfulness;S3=sigh;S4=stool abnormity;S5=dyssomnia;S6=weakness;S7=pant;S8=stomach distension;S9=chest distress;S10=palpitation;S11=soreness of waist.
